# Comparing wastewater-based and case-based *R_t_* estimates of SARS-CoV-2 transmission in Georgia using generalized linear mixed models

**DOI:** 10.1017/S0950268826101356

**Published:** 2026-04-06

**Authors:** Seth Edmunds, Douglas Landsittel, Marco Ajelli, Maria Litvinova

**Affiliations:** 1 Department of Epidemiology and Biostatistics, Indiana University - Bloomington School of Public Health, USA; 2 Laboratory for Computational Epidemiology and Public Health, Indiana University - Bloomington School of Public Health, USA; 3 Department of Biostatistics, State University of New York at Buffalo, USA

**Keywords:** wastewater surveillance, Reproduction number, GLMM, SARS-CoV-2, Georgia

## Abstract

The COVID-19 pandemic has highlighted limitations in case-based surveillance due to inconsistent testing and reporting. Wastewater-based epidemiology (WBE) has emerged as a complementary surveillance approach for tracking SARS-CoV-2 transmission, capturing both symptomatic and asymptomatic infections. The aim of this study was to evaluate the effectiveness of WBE in estimating the effective reproduction number ( 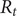 ) of SARS-CoV-2 in Georgia, USA. We used a Generalized Linear Mixed Model (GLMM) to analyse viral concentration data from multiple wastewater treatment plants (WWTPs) collected between 1 June 2022 and 15 December 2022. After controlling for flow rates and site-level heterogeneity, model residuals were transformed into a non-negative incidence-like series used to estimate wastewater-based 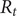 . Wastewater-based 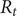 was compared with case-based 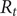 estimates using Spearman correlation. The two 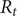 estimates showed concordant temporal patterns across most sites, with stronger correlations in areas with higher case counts (Spearman correlations ranging from 0.39 to 0.84, 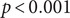 ). Wastewater-based 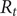 tracked increases and decreases in transmission over similar time scales as case-based estimates, while exhibiting reduced sensitivity to short-term changes in clinical testing and reporting behaviour. These findings suggest that WBE can support estimation of transmission trends and complement traditional case-based surveillance for public health monitoring.

## Introduction

The COVID-19 pandemic has underscored the need for effective surveillance systems to monitor disease transmission at the population level. Initially, case-based surveillance was critical for tracking the spread of COVID-19. However, fluctuations in testing rates and changes in reporting practices gradually undermined its reliability. By late 2022, the shift by the Centers for Disease Control and Prevention (CDC) from daily to weekly aggregated case and death reporting reduced the temporal resolution of data available for public health monitoring. This change was intended to reduce the reporting burden and improve operational efficiency, but it limited the ability to assess localized and short-term transmission dynamics. After the expiration of the public health emergency on 11 May 2023, the U.S. Department of Health and Human Services (HHS) could no longer require laboratories to submit COVID-19 testing results; subsequently, hospital COVID-19 reporting requirements were discontinued effective 1 May 2024 [1]. Together, these changes highlight the need for complementary surveillance approaches capable of monitoring population-level transmission when clinical reporting is incomplete or delayed.

In response to these challenges, wastewater-based epidemiology (WBE) has re-emerged as a promising complementary approach, providing a non-invasive, scalable method to track viral RNA in community wastewater, capturing both symptomatic and asymptomatic infections. WBE offers a unique opportunity to supplement traditional case-based surveillance, particularly by providing a population-level signal that is less dependent on individual testing behaviour and can reflect changes in community transmission. Because wastewater surveillance occurs at the community level, it is less sensitive to biases arising from uneven access to testing and underreporting, making WBE especially valuable in settings with limited healthcare access or inconsistent clinical testing [2, 3].

With the rise of at-home COVID-19 testing, which is largely not reported, comparing wastewater-based and clinical estimates of the effective reproduction number ( 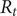 ) has become increasingly important for understanding transmission dynamics while recognizing that each data stream reflects different processes and sources of spatio-temporal heterogeneity. Recent studies have begun to leverage wastewater data alongside clinical surveillance to infer transmission dynamics and develop early-warning systems under varying assumptions about shedding, reporting, and observation processes [4]. By capturing SARS-CoV-2 RNA at the community level, WBE provides a population-scale signal that is less influenced by biases in clinical data, such as uneven testing access, changing individual behaviour, or reporting delays, though it is subject to its own sources of variability [5].

Several studies have estimated wastewater-based SARS-CoV-2 effective reproduction numbers using various assumptions, preprocessing, and model complexities [2, 6]. While county-level estimations have shown high concordance with traditional case-based estimates [7], there is a need to validate these methods at finer scales. Using a unique dataset that includes both reported cases and wastewater surveillance at the level of individual sewersheds, we demonstrate the concordance of 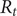 estimations on a disaggregated level. This study provides evidence for the operational utility of wastewater-based 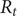 estimations and offers a practical pipeline accounting for sewershed-related heterogeneity.

The aim of this study was to evaluate the effectiveness of WBE in estimating 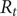 of SARS-CoV-2 at a sewershed level. We focus on data collected between 1 June and 15 December 2022 – a period during which routine case reporting via electronic laboratory and provider reports remained available, enabling a contemporaneous comparison of wastewater-based and case-based 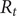 estimates. To compare these signals, we apply a Generalized Linear Mixed Model (GLMM) to wastewater concentrations, adjusting for temporal trends, flow-related dilution, and persistent sewershed differences. This approach allows us to assess the degree to which wastewater-derived transmission dynamics align with contemporaneous case-based 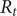 estimates at the sewershed level, and to evaluate the role of WBE as a complementary surveillance signal for clinical data at finer scales [1, 8].

## Methods

### Data collection

#### Wastewater data sources

WWTP employees collected twice-weekly wastewater samples, which were shipped to two university laboratories for analysis. Sampling frequency was typically twice weekly across sites, though exact schedules varied by facility and operational constraints (summarized in Supplementary Table A1). Viral RNA concentrations were quantified using PCR, specifically targeting the N2 gene for its widespread use and comparable performance to the N1 target (Huisman et al. [2]). RT-qPCR was used at WWTP 9 following the method detailed by Lott et al. [9] due to logistical constraints, while dPCR, preferred for its higher sensitivity to low viral concentrations, was used at all other sites following the protocol described by Sablon et al. [10]. Differences in analytical platform across sites were treated as time-invariant site characteristics and accounted for through site-level random effects in downstream modelling. Flow rate data were obtained from routine facility records and aligned with wastewater sampling dates; depending on facility practice, measurements corresponded to composite (time- or flow-weighted) or grab sampling approaches, consistent with standard wastewater surveillance reporting practices [11]. Concurrent flow rates were incorporated to account for dilution-related variability in wastewater volume.

#### Case data sources

Case data were obtained from routine provider and electronic laboratory reports submitted to the Department of Public Health (DPH) and stored within an electronic disease reporting system. Residential addresses were geocoded to obtain XY coordinates. Sewershed shapefiles were provided by the facilities. Case-to-sewershed assignment assumes accurate residential geocoding and alignment with sewershed boundaries, consistent with standard public health surveillance practice. Case data were aggregated daily by sewershed geography using symptom onset date when available; specimen collection date was used otherwise.

### Data processing

#### Case data processing

Cases were assigned to sewersheds based on geocoded residential addresses and the spatial overlap with sewershed boundaries. Epidemic curves were generated using either the date of symptom onset or, if unavailable, the date of the first positive specimen (including asymptomatic cases). Missing daily case counts were imputed using Kalman filtering to provide smoothed estimates, ensuring the preservation of temporal sequences while handling reporting fluctuations. Imputation primarily reflects completion of a daily time series from routine non-daily reporting rather than filling extended gaps in surveillance, and the Kalman approach was selected based on comparative performance in preserving temporal structure, as summarized in the Supplementary Material. Data were restricted to the study period from 1 June 2022 to 15 December 2022, and the processed case data were used in conjunction with wastewater viral concentrations for analysis.

#### Wastewater data processing

All data are from eight sewersheds in Georgia, USA. Outliers in wastewater viral concentrations and flow rates were detected using the interquartile range (IQR) method, with outliers replaced by the median to reduce their influence on downstream analysis. Flagged values were treated as transient spikes for robustness rather than assumed measurement errors, and the number of flagged observations by site is reported in Supplementary Table A2. While Supplementary Table A1 summarizes the characteristics of all 10 facilities participating in the broader state surveillance programme, two facilities, WWTP 4 and WWTP 7, lacked sufficient data during the study period window (1 June –15 December 2022). Because only observations exceeding the IQR threshold were modified, minimum and maximum values could remain unchanged if those extremes did not meet the outlier criterion. This method was selected based on a sensitivity analysis comparing multiple outlier detection techniques, including median absolute deviation (MAD), using agreement with case-based 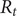 (Spearman correlation and RMSE) as evaluation criteria. Samples were predominantly 24-h composite, collected twice weekly. Population served by individual sewersheds varied from 10000 (WWTP 10) to 650000 (WWTP 1). Sewershed locations spanned urban, suburban, and rural areas across Georgia; specific geographic coordinates are not disclosed to protect facility identities, but site-level population, flow, and sample characteristics are summarized in Supplementary Table A1. Concentrations were not pre-normalized. Instead, by including flow as a fixed effect in the GLMM, the model inherently adjusts for flow-related dilution, with the resulting residuals representing viral signal fluctuations independent of flow variation.

Preprocessing method selection was guided by comparative evaluation across multiple approaches. Outlier detection using the IQR method flagged 27 concentration values and 57 flow values across sites, compared with 27 and 123, respectively, for the MAD approach. Missing data imputation using Kalman filtering (71.8% of the daily time series) reflected completion to daily resolution from approximately twice-weekly sampling; inter-sample gaps had a median of 2 days. Sensitivity analyses across rolling window lengths showed that 14-day windows achieved the highest concordance with case-based 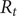 estimates (median Spearman *ρ = 0.608*, IQR = 0.121), comparable to 10-day windows (*ρ = 0.612*, IQR = 0.184) and superior to 7-day windows (*ρ = 0.593*, IQR = 0.254). The selected preprocessing combination (IQR outlier detection, Kalman imputation, 14-day rolling average) balanced noise reduction with preservation of transmission trends and minimized data loss (flagging fewer than half as many flow outliers as the MAD approach), as assessed by correlation with case-based estimates (median RMSE = 0.098). Complete site-level characteristics, outlier counts, missingness patterns, and sensitivity results are provided in Supplementary Tables A1–A4.

Missing data were interpolated using Kalman filtering, chosen for its performance relative to linear and spline interpolation methods in preserving temporal structure during routine non-daily sampling [12, 13]. Both viral concentrations and flow rates were log-transformed to stabilize variance and normalize the data distribution. Comparisons of raw and processed data are presented in Supplementary Table A5. These preprocessing steps were implemented to stabilize the time series for downstream transmission trend estimation rather than to reconstruct true incidence or shedding processes.

### Statistical modelling

#### Model specification

A GLMM was employed (significance level set to 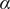 =0.05) to analyse viral concentrations in wastewater, with both fixed and random effects to capture the hierarchical and temporal structure of the data. The fixed effects included time and flow rate, while the random effects accounted for site-level variability across WWTPs. Model diagnostics were used to assess statistical fit and stability of the mixed-effects model, including residual behaviour and random-effect variance. While these diagnostics ensure the statistical robustness of the framework, they do not, by themselves, imply the biological validity of the resulting residual time series as a direct and comprehensive measure of the number of infections. By accounting for both site-specific and time-based variability, the model provided a flexible framework for analysing the transmission dynamics signal [14, 15].

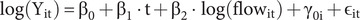

Where:

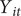
 is the viral concentration in wastewater at time 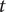 for WWTP 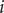 .

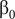
 is the fixed intercept representing the global baseline concentration.

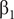
 is the coefficient for the linear temporal trend.

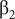
 is the coefficient adjusting for the log-transformed wastewater flow rate.

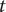
 is a daily time variable to account for temporal trends.

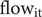
 is the flow rate of wastewater at time 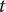 for WWTP 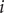 .

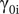
 is the random intercept for WWTP 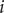 , capturing time-invariant site-specific differences where 
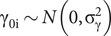
 .

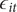
 is the residual error term.

The primary specification includes a fixed effect for temporal trend and a site-specific random intercept, implying a common temporal trend with persistent site-specific differences. By utilizing a GLMM instead of manual flow-normalization, we provide a unified framework to adjust simultaneously for flow-related dilution, shared temporal trends, and site-level heterogeneity (e.g., variations in measurement and sampling approaches) that are captured by the random intercept ( 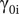 ). By intentionally omitting site-specific random slopes for time, we ensure that localized deviations from the regional trend are preserved within the residuals ( 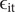 ) rather than being absorbed into the model parameters. This approach allows the residuals to more accurately reflect the site-level dynamics necessary for subsequent 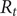 estimations while accommodating the nested data structure.

It is important to note that the linear time covariate is specified as a global fixed effect across a geographically diverse set of sewersheds. It is intended to control for the shared baseline trend without absorbing localized epidemic waves. However, researchers applying this framework should note that if applied to highly synchronized, homogeneous sites over a short period, the global time effect may absorb the epidemic signal, especially during the period of consistent epidemic growth or decline. In such cases, the approach should be implemented without the time trend fixed effects. Empirical demonstrations of the model’s robustness across varying observation windows and distinct transmission phases are provided in Supplementary Figures A1 and A2 respectively.

#### Residual extraction and smoothing

Residuals 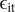 from the GLMM were extracted to capture variability in viral concentrations not explained by the fixed effects of time, flow rate, and site-specific differences in wastewater concentrations, including measurement differences. The inclusion of site-specific random intercepts accounts for persistent between-site differences in baseline viral concentration, allowing within-site temporal deviations to be examined on a comparable scale. To reduce high-frequency noise, a 14-day rolling average was applied to the residual series. While rolling averages introduce additional temporal smoothing and may attenuate short-term changes, this window length reflects a pragmatic balance between noise reduction and trend interpretability and was evaluated through sensitivity analyses reported in Supplementary Table A4. This approach ensures that critical transmission patterns are preserved while minimizing the impact of daily fluctuations. Accordingly, residual-based analyses are intended to support inference on transmission trends rather than to reconstruct individual-level infection, shedding processes, case, or hospitalization incidence.

#### Estimation of the effective reproduction number (*R*_*t*_)

The effective reproduction number ( 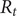 ) was estimated using the EpiEstim package in R [16, 17]. A parametric serial interval distribution was specified with a mean of 2.75 days and a standard deviation of 2.53 days, utilizing updated parameters specific to the SARS-CoV-2 Omicron variant (Kremer et al. [18]). To align with the requirements for 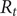 estimation, the GLMM residuals were exponentiated to back-transform the values from the log-link model and ensure the non-negativity required for an incidence proxy. Two estimates of 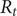 were calculated: one based on case-derived incidence, and one based on a transformed wastewater-derived residual series. Both case-based and wastewater-based series were smoothed using a 14-day rolling average prior to 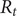 estimation to reduce short-term noise, with the understanding that this additional smoothing further convolves the underlying signal; the impact of window length on 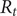 concordance was evaluated through sensitivity analyses reported in the Supplementary Table A4 [2, 3]. As a result, wastewater-derived 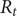 estimates are interpreted as a signal of relative transmission dynamics rather than as direct estimates of intrinsic incidence-based reproduction parameters.

A sensitivity analysis was performed to assess the impact of rolling average window length on both case-based and wastewater-based 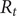 estimates. Rolling windows ranging from 3 to 30 days were evaluated, with performance assessed based on agreement with case-based 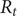 (Spearman correlation and RMSE) and qualitative stability of inferred trends. The 14-day window was selected for primary analysis because it provided a pragmatic balance between noise reduction and temporal responsiveness across sites, as summarized in Supplementary Table A4. Longer windows further reduced noise but increasingly attenuated short-term changes during periods of rapid transmission change [19].

To quantify concordance between wastewater-based and case-based 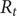 estimates, we computed Spearman rank correlation coefficients across sites. Analyses were restricted to the period prior to December 2022, after which changes in case reporting requirements substantially altered the reliability and completeness of case-based surveillance. Alignment was further assessed by comparing the temporal co-movement of wastewater-based 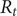 , case-based 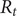 , and reported case counts, focusing on direction and timing of major transmission changes rather than pointwise equality. This approach emphasizes concordance in transmission trends under differing observation processes rather than validation against a presumed ground truth [20].

## Results

The initial dataset comprised 294 observations for both PCR target average concentration and flow rate measurements across eight WWTPs. After data preprocessing, which included outlier handling and missing data imputation, the number of observations increased to 1019. Detailed statistics on preprocessing outcomes are presented in the Supplementary Material. A total of 749 data points (72% of the dataset) were imputed using Kalman smoothing, reflecting routine completion of a daily time series from non-daily (typically twice-weekly) sampling rather than extended gaps in surveillance. Kalman smoothing was employed because it effectively estimates missing values in time series data by modelling the underlying stochastic processes. This method accounts for variability by incorporating both the observed data and the process noise, allowing for smoothed estimates of the underlying temporal signal amidst measurement uncertainty and routine sampling variability [13].

**Figure 1.**
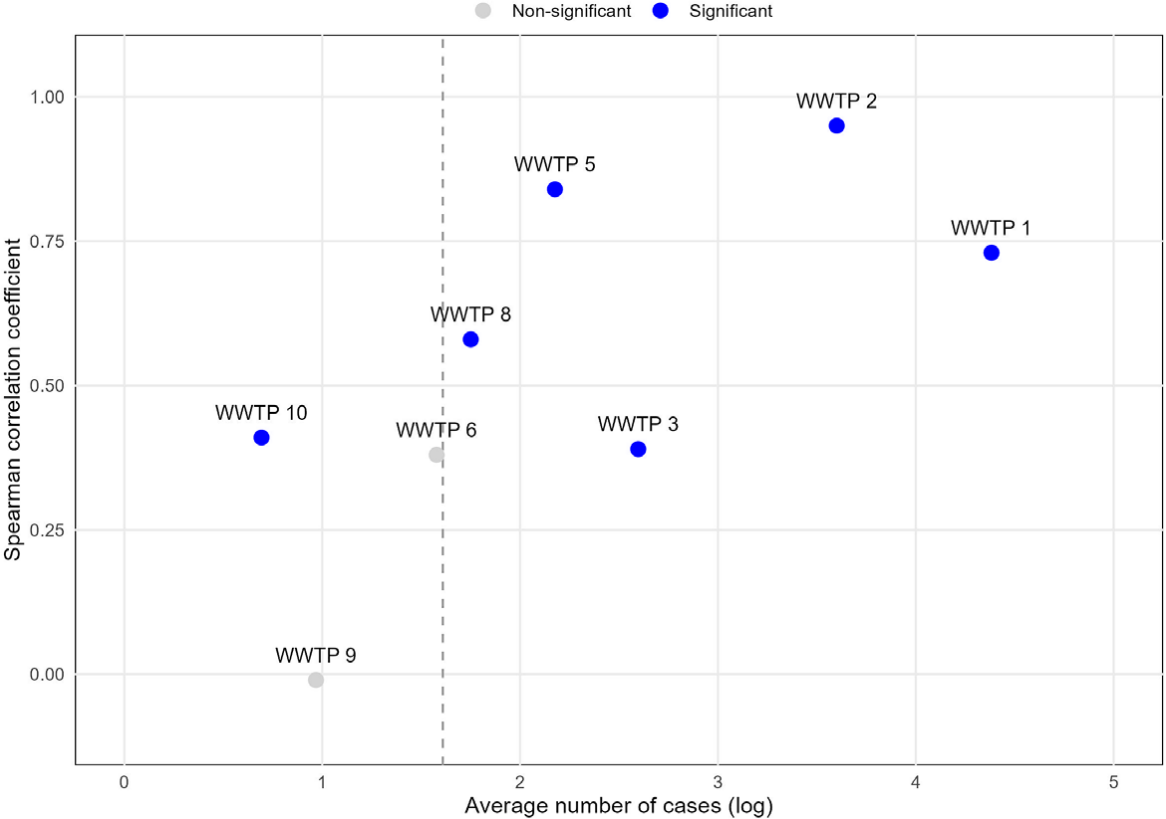
Average number of cases and Spearman correlation coefficient between case-based and wastewater-based 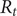 estimates by the wastewater treatment plant’s sewershed.

An overview of the 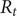 estimates derived from both case-based and wastewater-based surveillance is provided in Table 1. This table includes the duration of data collection, total case counts, and median 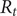 values, and Spearman correlation coefficients across the eight wastewater treatment plants (WWTPs). All statistically significant results illustrate a notable positive correlation between the two 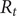 estimations, with the highest correlation being observed for WWTP 5 (0.84, 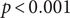 ) and the lowest for WWTP 3 (0.39, 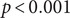 ). The results (Table 1) indicate that average case burden influenced both correlation strength and statistical significance (Figure 1). For example, no statistically significant correlation was observed for WWTP 9; while its data collection duration was comparable to WWTP 1, its average case burden was substantially lower. Generally, correlation strength and statistical significance aligned with overall case burden. Sewersheds with very low daily case counts frequently lacked the temporal variability required to power rank-based correlation (e.g., WWTP 9 and WWTP 6). However, statistically significant correlations were occasionally observed even in low-incidence settings (e.g., WWTP 10, averaging approximately 2 cases per day). The overall pattern is illustrated in Figure 1, where 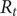 concordance generally increases as a function of the average number of cases.

**Table 1.** Core statistics of 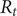 Estimates, correlations, and sewershed characteristics (ordered by descending case burden)

Sewershed	Duration (days)	Total cases	Median *R*_*t*_ (Case)	Median *R*_*t*_ (WW)	Spearman correlation	P-value
WWTP 1	191	15288	1	1	0.73	*<0.001*
WWTP 3	191	2564	1	1	0.39	*<0.001*
WWTP 5	177	1560	1.02	1.03	0.84	*<0.001*
WWTP 8	191	1100	1.02	1.03	0.58	*<0.001*
WWTP 2	26	952	1.11	1.17	0.95	*<0.001*
WWTP 9	191	503	1.06	1.02	−0.01	*0.841*
WWTP 6	26	126	1.06	1.23	0.38	0.052
WWTP 10	26	52	1.06	1.2	0.41	*0.035*

The vertical dashed line indicates approximately five average daily cases (log scale), below which rank-based correlation was generally underpowered in our data, though exceptions were observed (e.g., WWTP 10).

### Overview of *R*_*t*_ estimates across all sites

Table 2 illustrates the percentage of days on which the lower and upper bounds of the 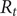 confidence intervals (CIs) were either above or below the epidemic threshold (1.0). This metric provides supplementary information on directional agreement relative to the epidemic threshold ( 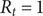 ), but was not used as a primary performance criterion because it is sensitive to smoothing choices and uncertainty width.

**Table 2.** Percent of days with 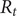 confidence intervals above or below 1 (ordered by descending case burden)

Sewershed	Case-based				Wastewater-based			
	95% CI		50% CI		95% CI		50% CI	
	> 1	< 1	> 1	< 1	> 1	< 1	> 1	< 1
WWTP 1	3.14	17.28	20.94	28.8	25.13	7.85	43.46	33.51
WWTP 3	0.52	0	9.95	12.04	12.57	12.57	37.7	30.89
WWTP 5	0.56	0	14.69	10.17	31.64	15.82	54.24	33.9
WWTP 8	0	0	4.71	4.19	8.9	8.38	49.74	27.75
WWTP 2	19.23	0	96.15	0	84.62	0	100	0
WWTP 9	0	0	8.38	0	2.09	0	40.84	8.38
WWTP 6	0	0	7.69	0	88.46	0	96.15	0
WWTP 10	0	0	7.69	0	65.38	15.38	73.08	19.23

## Detailed analysis of representative sites

The wastewater-based and case-based 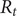 estimates were analysed across five representative sites with total case incidence greater than 1000 cases during the study period. Each site exhibits unique patterns in transmission dynamics, as captured by the two surveillance methods. Sites were selected to represent higher-incidence sewersheds where case-based 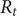 estimates showed sufficient variability to support meaningful temporal comparisons. The sites are presented in the order of decreasing case burden. Two levels of uncertainty around median estimates (50% and 95% CIs) are used to represent two levels of potential risk tolerance of public health practitioners in making decisions based on uncertain data.

**Figure 2.**
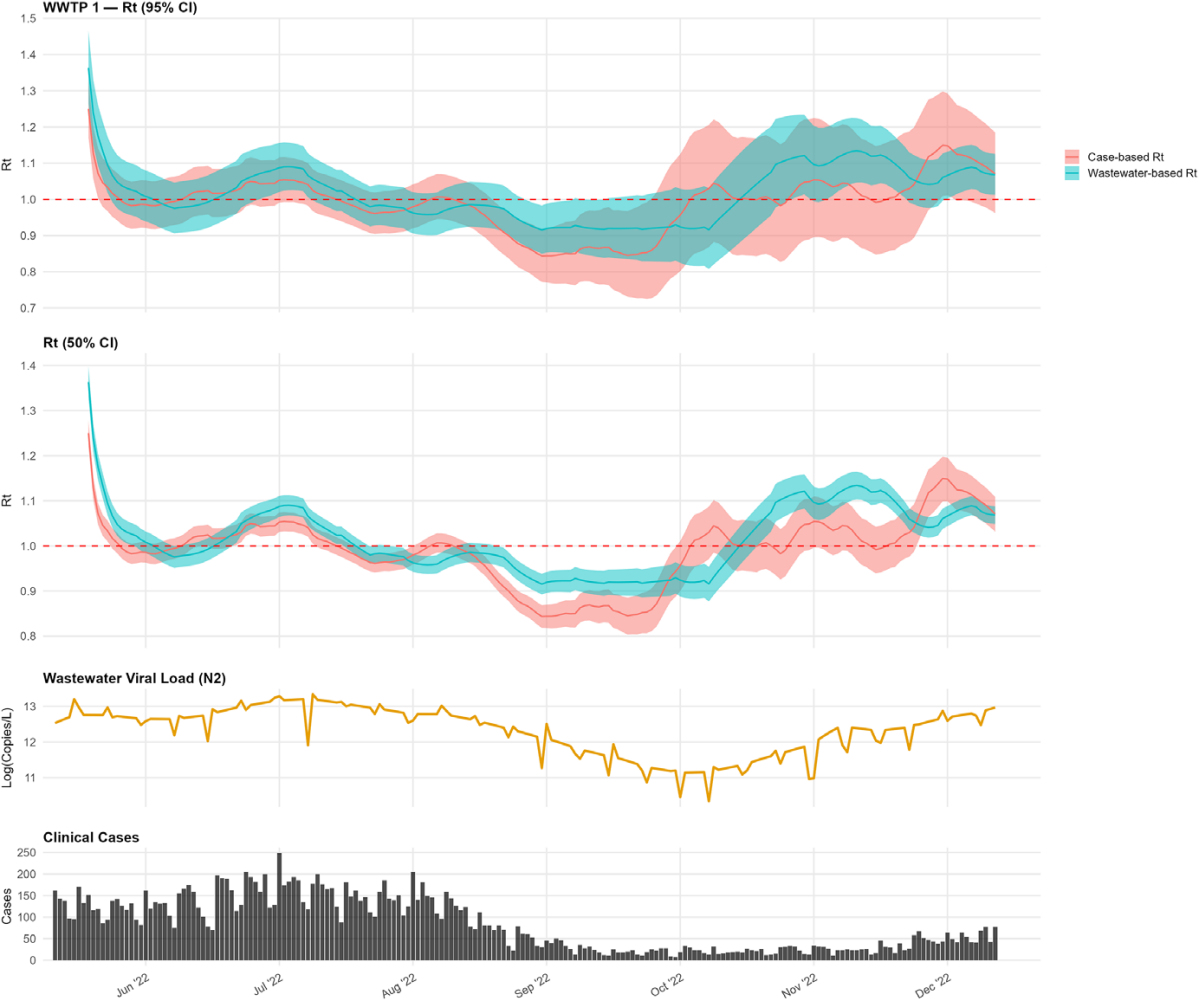
Comparison of wastewater-based and case-based 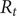 estimates for WWTP 1.

At WWTP 1, the wastewater-based and case-based 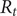 estimates closely tracked from June through August, both peaking in mid-July, which aligns with the peaks in the interpolated wastewater signal and clinical case incidence (Figure 2). By late August, both estimates fell below the epidemic threshold ( 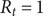 ). From mid-October onward, the wastewater-based estimate climbed above the epidemic threshold earlier than the case-based estimate in this sewershed.

**Figure 3.**
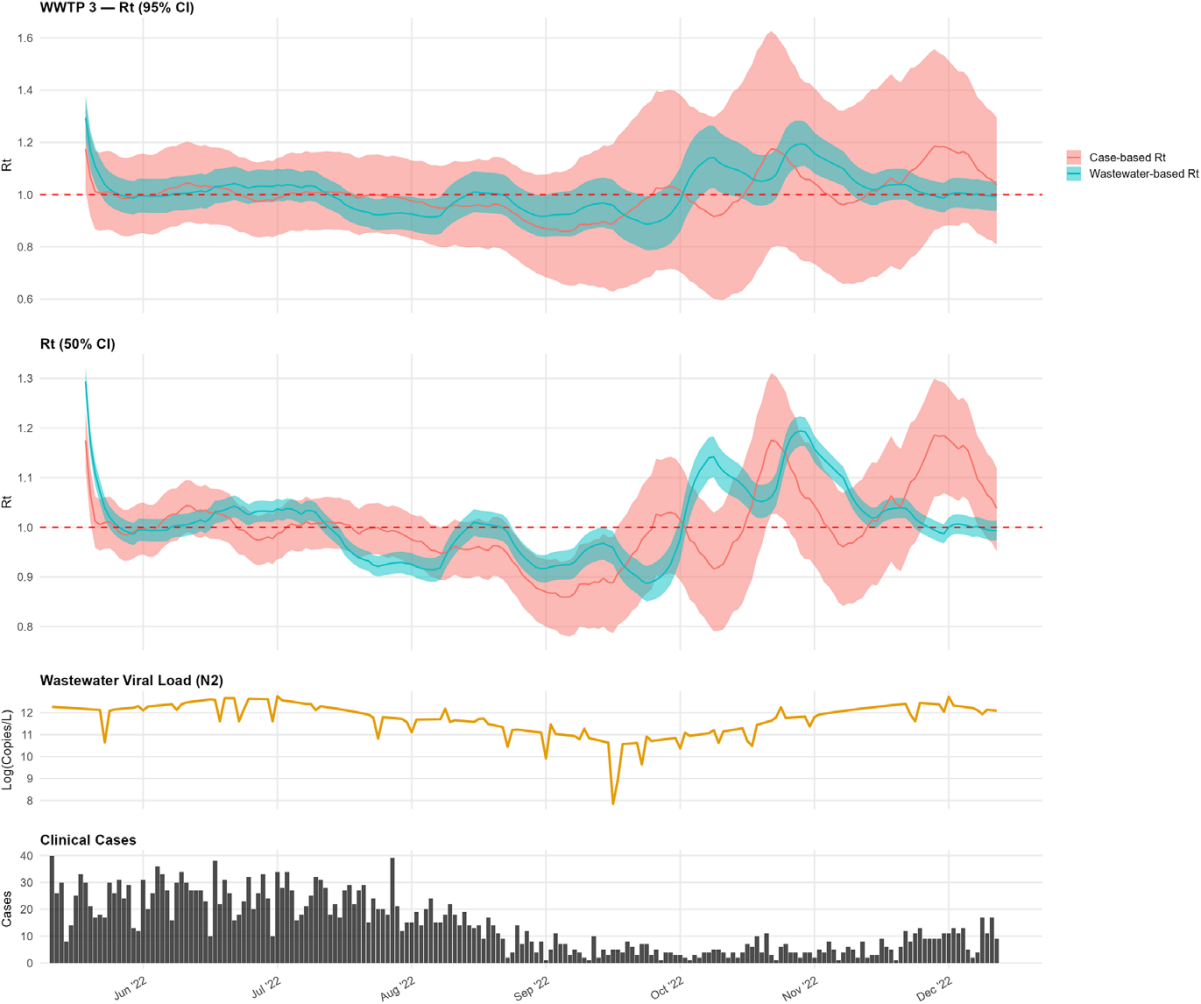
Comparison of wastewater-based and case-based 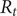 estimates for WWTP 3.

In WWTP 3, the wastewater-based estimates sometimes lagged peaks in the case-based 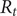 (Figure 3). In mid-October, the wastewater-based 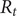 increased to approximately 1.1–1.3, with comparatively narrower CIs, while the case-based estimates exhibited wider uncertainty that encompassed the threshold of 1.0.

**Figure 4.**
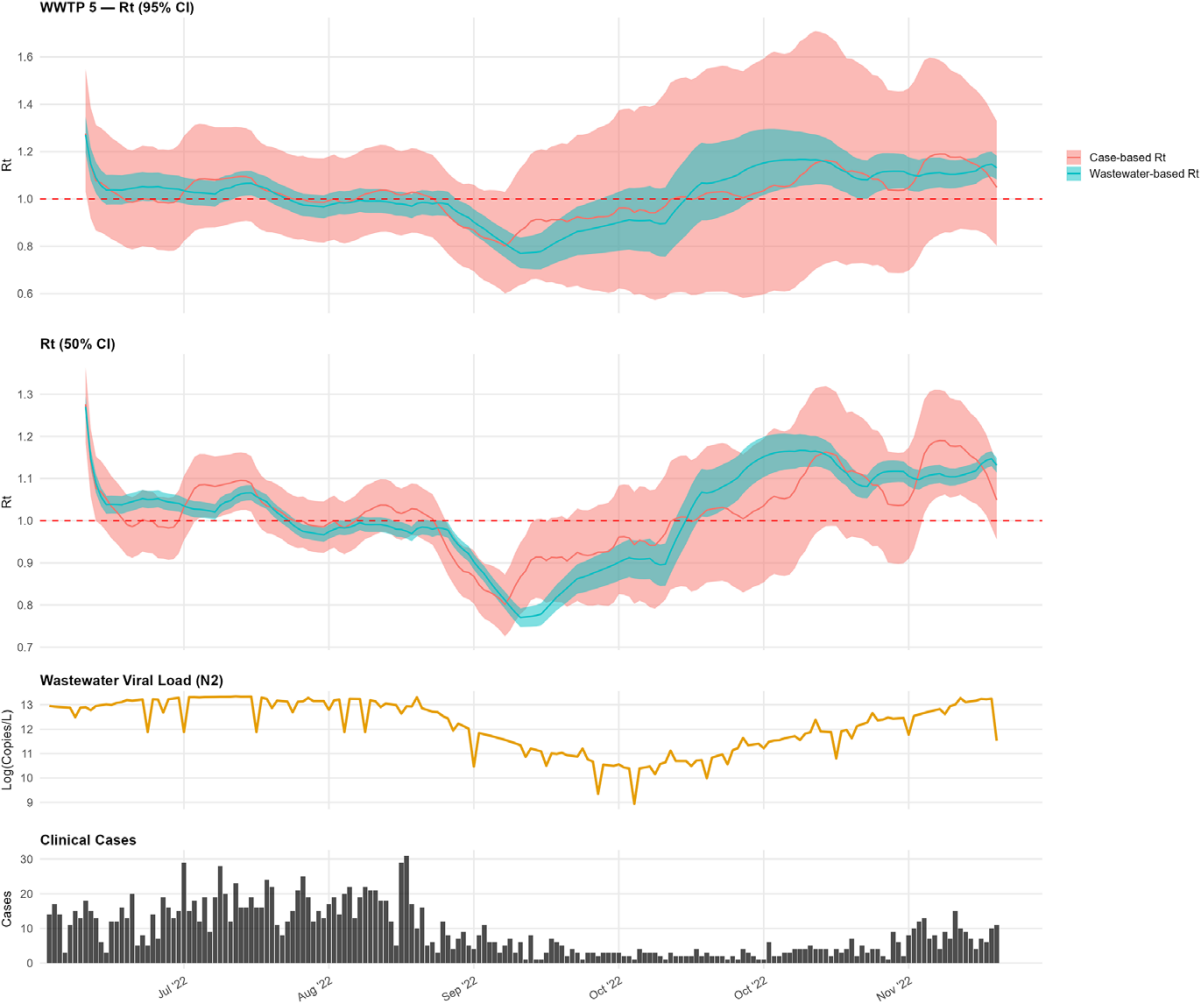
Comparison of wastewater-based and case-based 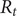 estimates for WWTP 5.

For WWTP 5, the wastewater-based 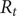 initially aligned with case-based trends during early summer, with values above 1.0 (Figure 4). In mid-September, the wastewater-based 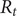 declined below 0.8 prior to a similar decline in the case-based estimate. A subsequent increase in the wastewater-based 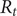 in mid-October preceded a rise in cases.

**Figure 5.**
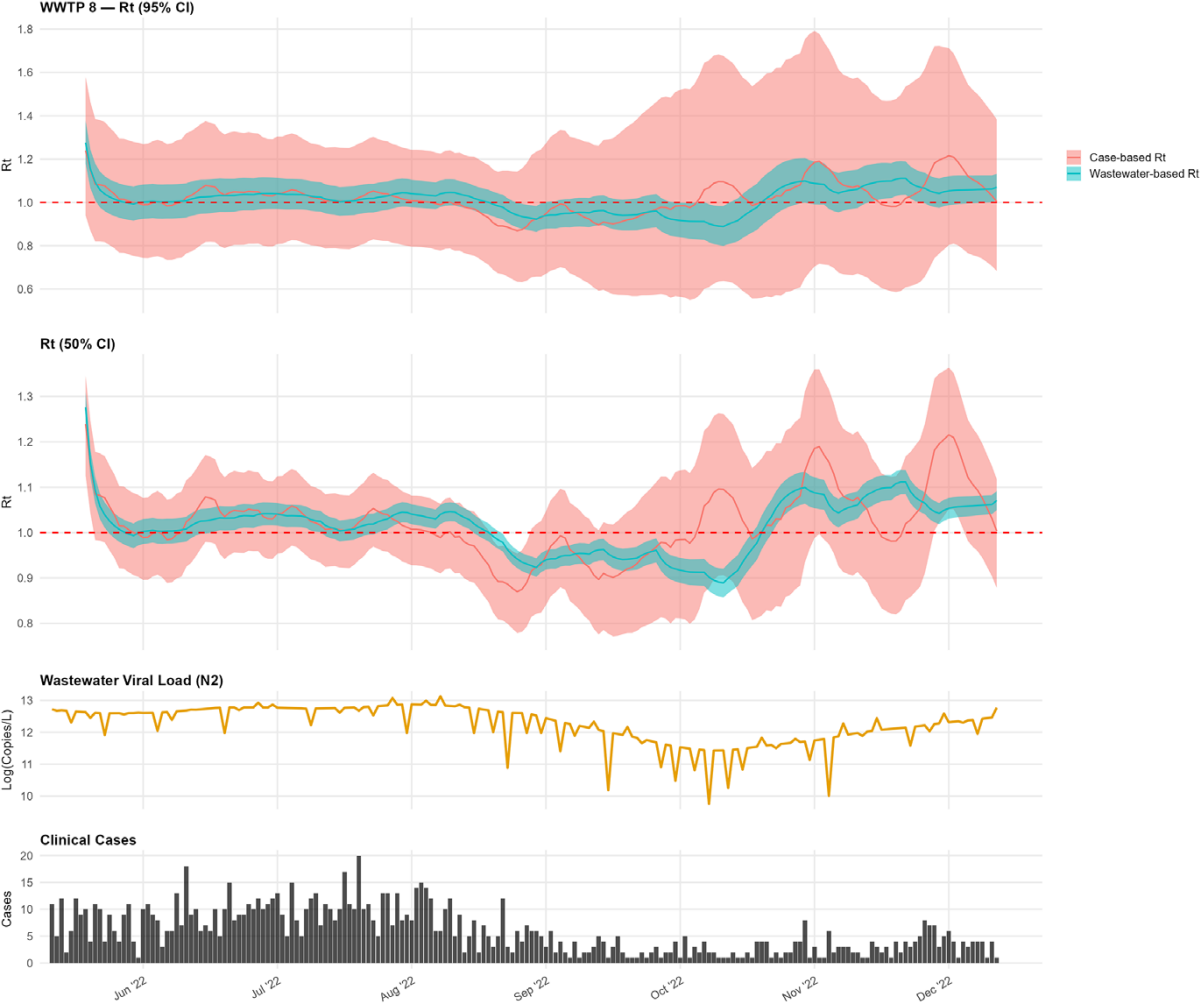
Comparison of wastewater-based and case-based 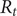 estimates for WWTP 8.

At WWTP 8, the wastewater-based 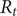 remained above 1.0 from early July, with brief dips (Figure 5). Case-based 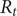 exhibited broadly similar temporal patterns but with shifts in timing consistent with differences in smoothing and observation processes.

**Figure 6.**
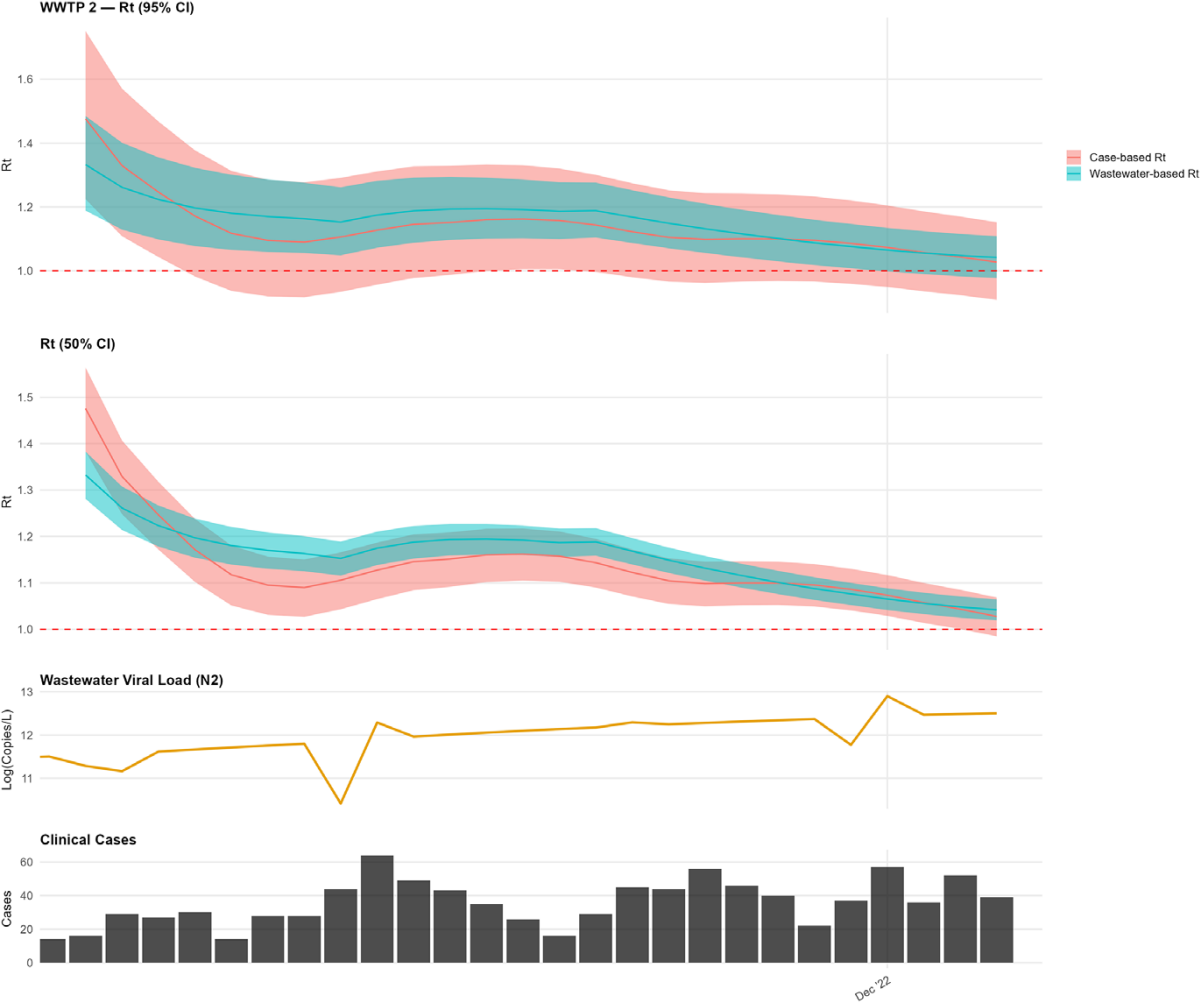
Comparison of wastewater-based and case-based 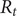 estimates for WWTP 2.

At WWTP 2, over the shorter study period, the wastewater-based 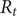 remained consistently above 1.0, while the case-based 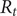 rose above 1.0 in early December (Figure 6). An initial elevation in the wastewater-based estimate was interpreted as a transient anomaly consistent with preprocessing and smoothing effects rather than sustained transmission change.

## Discussion

Our analyses indicate that wastewater-based 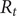 estimates exhibited broadly similar temporal patterns to case-based estimates across most sites, capturing transmission shifts within approximately 2 weeks of clinical data. The fundamental purpose of evaluating a clinical comparator is to determine whether pathogen detection in wastewater translates into a tangible public health burden. However, selecting an appropriate clinical comparator requires navigating the inherent limitations of all potential public health signals. Because 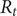 estimation relies on the ratio of successive incidence values rather than absolute counts; it remains mathematically robust to systematic underreporting provided ascertainment does not shift abruptly. Therefore, the optimal choice must be guided by the stability of the signal during the observation window and the specific practical needs of public health authorities.

For this study, we utilized reported case incidence by symptom onset or collection date. While such data inherently underestimates true infection burden, as demonstrated by infection incidence being 2.2–3.7 times higher than reported cases in the United States during the Omicron wave [21], reporting requirements during our study period (June–December 2022) were sufficiently stable to establish a reliable baseline. The subsequent expiration of public health mandates and the wider utilization of non-reportable at-home testing rapidly rendered clinical ascertainment lower and highly volatile [21], necessitating our choice of the study’s end date. Therefore, for this study, reported cases provided the most stable, geospatially appropriate comparator to address the local public health burden at the sewershed level. However, during periods of rapid surveillance infrastructure changes, divergence between wastewater and clinical signals should not be interpreted as a failure of wastewater surveillance, but rather as a complementary reflection of community transmission dynamics essential for comprehensive situational awareness.

Several limitations should be acknowledged. A substantial portion of the time series required completion to daily resolution for 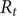 estimation, primarily reflecting routine non-daily sampling rather than prolonged data gaps. Kalman smoothing was used to interpolate missing days and stabilize temporal structure, which may attenuate short-term variability but is necessary for renewal-based estimation frameworks. Additionally, wastewater measurements reflect a convolution of infections over heterogeneous shedding durations and sewer transport processes. As a result, wastewater-derived 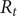 should be interpreted as a smoothed indicator of transmission trends rather than a direct measure of incident infections. This interpretation is subject to uncertainties and heterogeneities in SARS-CoV-2 shedding likelihood, rates, duration, and routes. Furthermore, while we account for the flow differences, variability introduced by site-specific shedding dynamics, viral variants, and in-sewer environmental factors impacts the precision of these estimates. Nevertheless, these uncertainties do not preclude the detection of major transmission shifts as evidenced by the strong concordance observed with case data in higher-burden areas.

Finally, assignment of COVID-19 cases to sewersheds relied on residential address geocoding, which may misclassify infections if substantial exposure or shedding occurred outside the residential sewershed. Future studies could enhance geolocation accuracy by incorporating additional data sources or methods, such as improved address resolution or alternative approaches to case-to-sewershed assignment. These limitations highlight areas for methodological improvement, but do not alter the central finding that wastewater-based 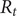 estimates captured transmission trends broadly consistent with case-based surveillance. Rather than enabling direct intervention decisions, wastewater-derived 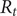 provides a complementary indicator of changing transmission dynamics that may support situational awareness when clinical surveillance is incomplete or delayed.

The weaker correlation observed at WWTP 3 (*ρ = 0.39*), despite higher total case counts compared with WWTP 5 (*ρ = 0.84*), likely reflects differences in case variability rather than absolute burden. WWTP 3 exhibited lower day-to-day variance in case incidence during the study period, reducing the discriminatory power of rank-based correlation metrics even when directional trends were broadly consistent.

Hill et al. [6] compared eight WBE-based 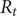 estimation approaches and found that methodological choices substantially influenced estimates. The present study does not seek to advance a competing method but rather to assess whether a relatively simple GLMM framework applied to routine twice-weekly sampling can yield transmission trend indicators concordant with case-based surveillance at the sewershed level.

Future research may benefit from evaluating how wastewater-based surveillance scales to larger geographic units, such as counties or clusters of WWTPs, and whether multiple facilities within a region capture consistent spatiotemporal transmission patterns. Assessing how wastewater-derived indicators can be aggregated without obscuring local dynamics would inform their use in regional surveillance frameworks. As wastewater surveillance continues to be integrated into public health practice, clarifying its role next to the traditional epidemiologic data will be essential for understanding how population-level signals can complement, rather than replace, case-based reporting systems.

## Supporting information

10.1017/S0950268826101356.sm001 Edmunds et al. supplementary material Edmunds et al. supplementary material

## Data Availability

Code used for this analysis is available at: https://github.com/sethmund/wastewater_glmm. Wastewater and case surveillance data are available upon request from the Georgia Department of Public Health, subject to data use agreements.

## Supplementary material

The supplementary material for this article can be found at http://doi.org/10.1017/S0950268826101356.
