# Supplementary figures and images for: Comparing wastewater-based and case-based Rt estimates of SARS-CoV-2 transmission in Georgia using generalized linear mixed models
Source: Epidemiol Infect. 2026 Apr 6;154:e63. doi: 10.1017/S0950268826101356 (PMC13184661; doi:10.1017/S0950268826101356)

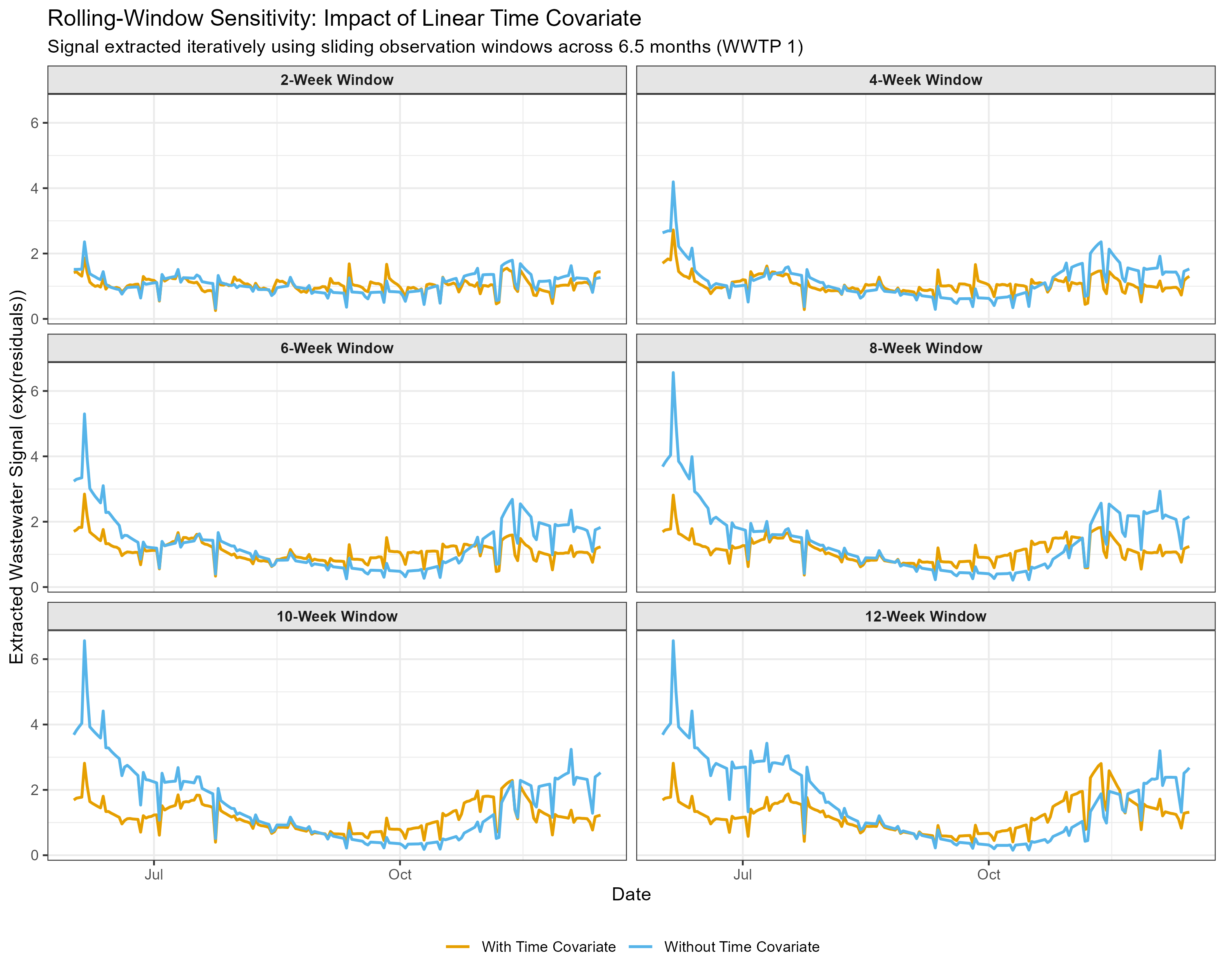

Supplement: Edmunds et al. supplementary material [file S0950268826101356sup001.zip › fig_A1_rolling_sensitivity.jpg]

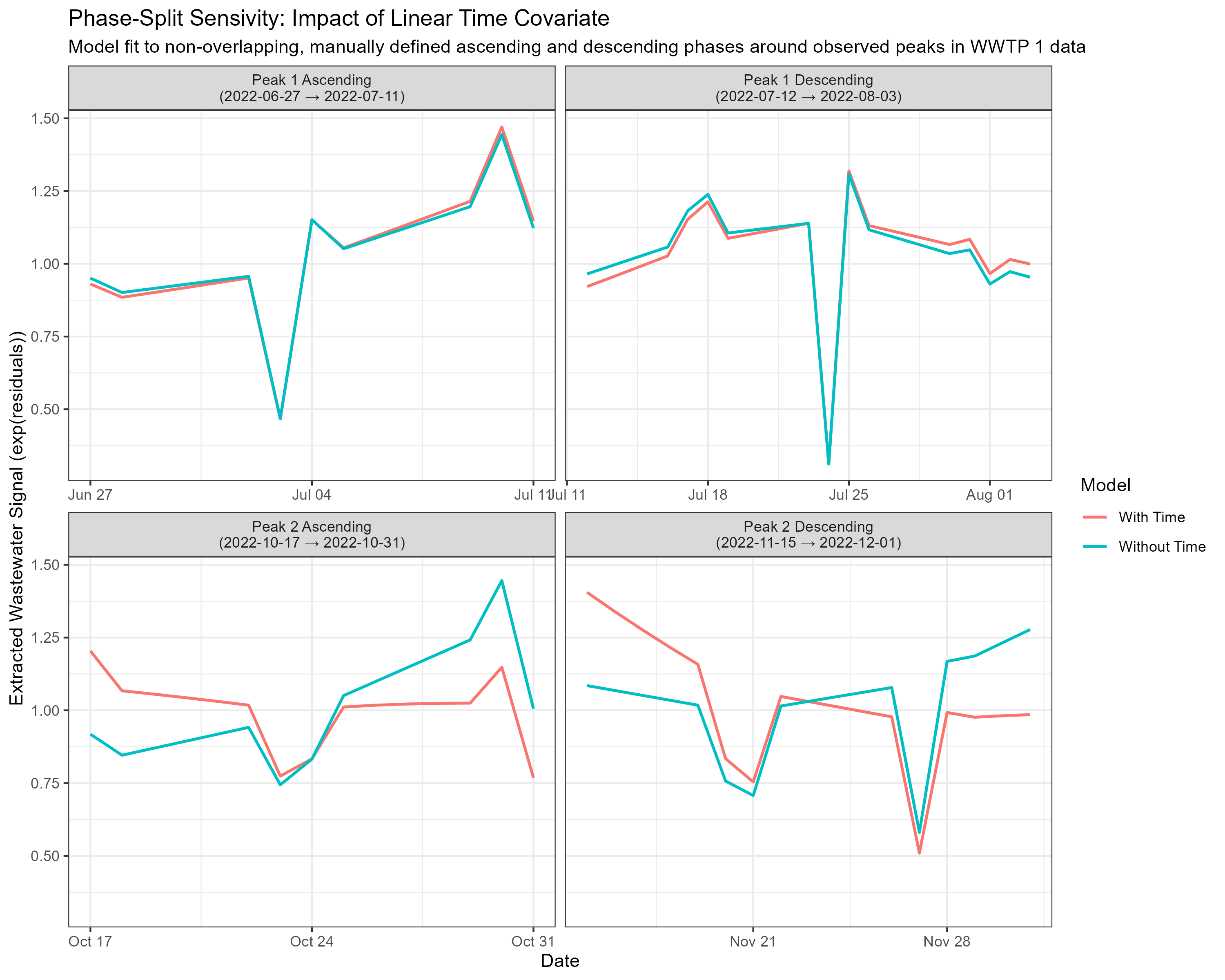

Supplement: Edmunds et al. supplementary material [file S0950268826101356sup001.zip › fig_A2_phase_sensitivity.jpg]

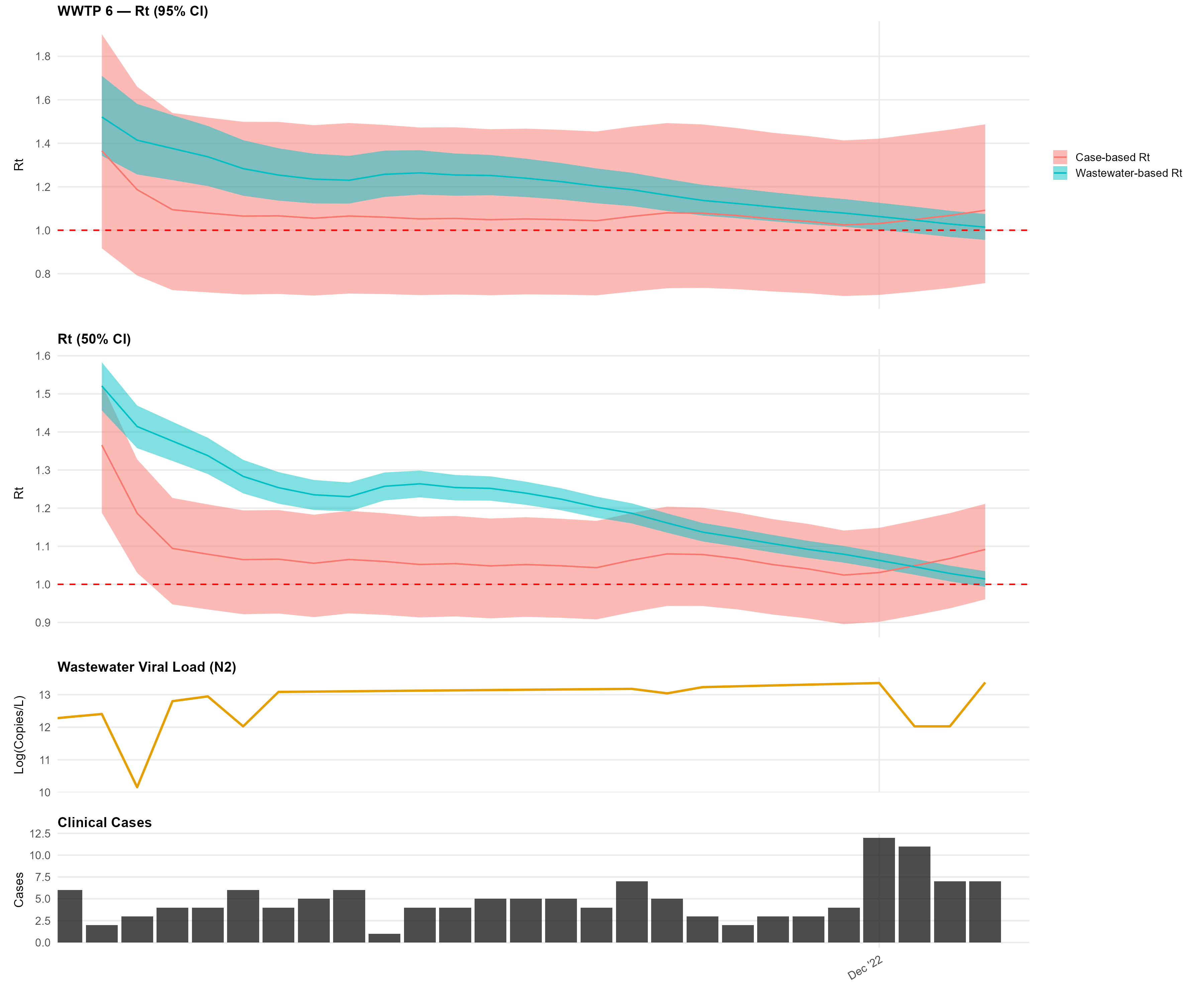

Supplement: Edmunds et al. supplementary material [file S0950268826101356sup001.zip › fig_A3_rt_comparison_WWTP 6.jpg]

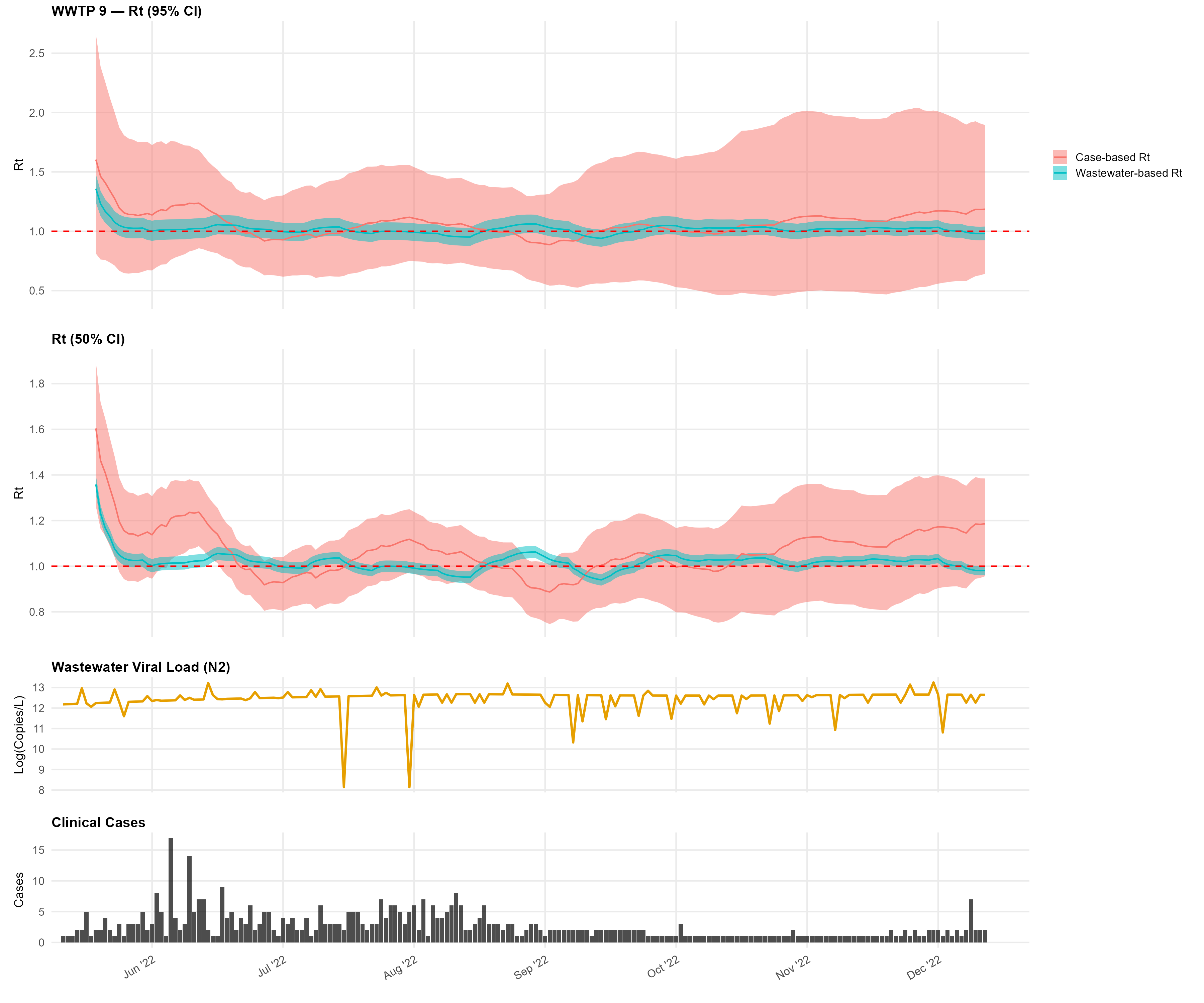

Supplement: Edmunds et al. supplementary material [file S0950268826101356sup001.zip › fig_A4_rt_comparison_WWTP 9.jpg]

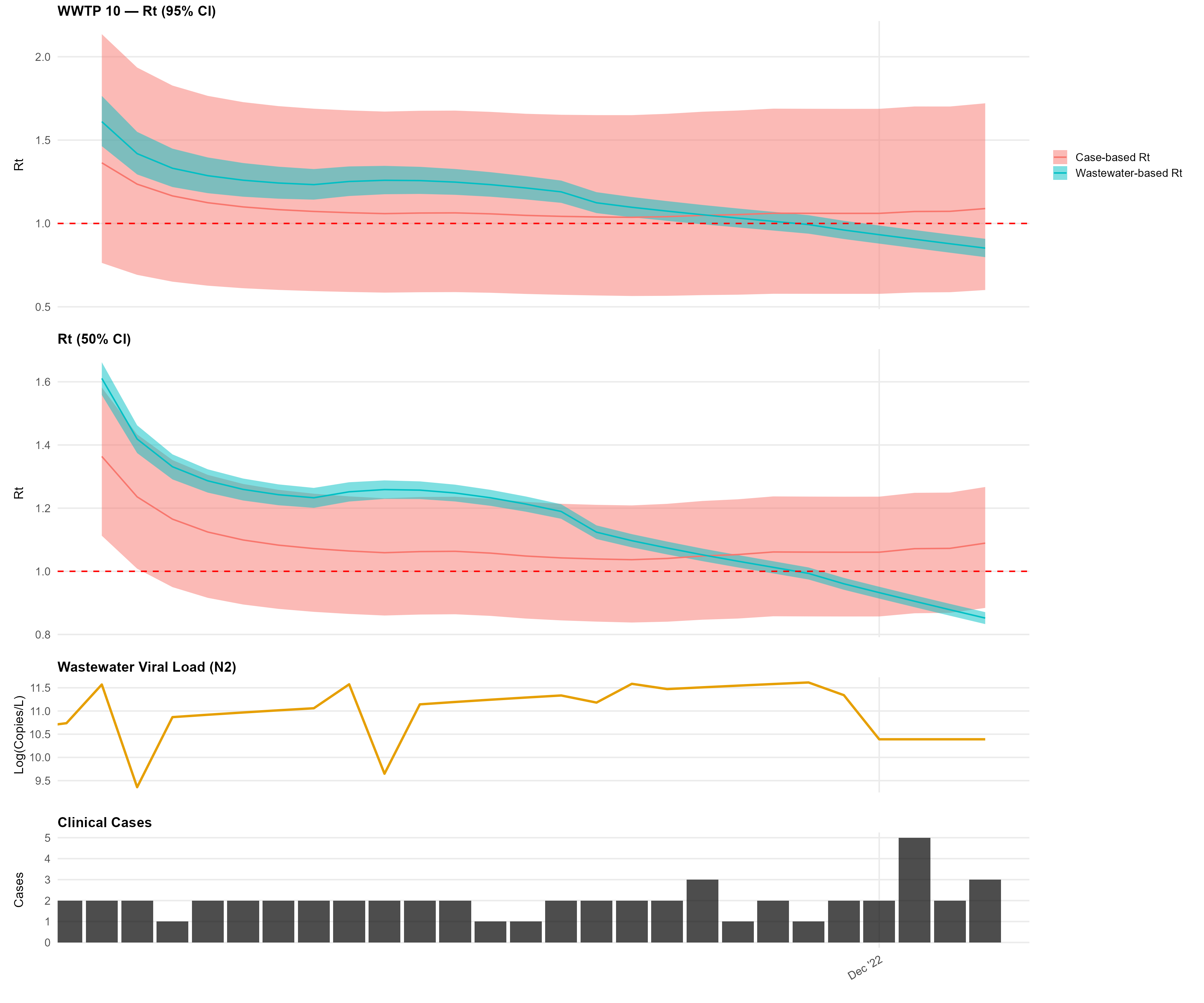

Supplement: Edmunds et al. supplementary material [file S0950268826101356sup001.zip › fig_A5_rt_comparison_WWTP 10.jpg]
